# Transcription at an inducible common fragile site reveals replication origin strength hierarchy

**DOI:** 10.1093/nar/gkag297

**Published:** 2026-04-07

**Authors:** Juliette Mandelbrojt, Caroline Tonnerre-Doncarli, Théo Baret, Aurélie Masson, Michelle Debatisse, Marie-Noëlle Prioleau

**Affiliations:** Université Paris Cité, CNRS, Institut Jacques Monod, F-75013 Paris, France; Université Paris Cité, CNRS, Institut Jacques Monod, F-75013 Paris, France; Université Paris Cité, CNRS, Institut Jacques Monod, F-75013 Paris, France; Université Paris Cité, CNRS, Institut Jacques Monod, F-75013 Paris, France; CNRS UMR 9019, Gustave Roussy Institute, F-94805 Villejuif, France; Sorbonne University, F-75005 Paris, France; Université Paris Cité, CNRS, Institut Jacques Monod, F-75013 Paris, France

## Abstract

While genome-wide analyses in vertebrates suggest that transcription negatively regulates replication origin activation, thereby shaping the landscape of replication initiation, it remains unknown whether efficient replication origins can overcome this suppression at common fragile sites (CFSs). In this study, we addressed this question by inserting two model origins into the avian DMD CFS and a transcriptionally silent late-replicating region in DT40 cells. The DMD gene, which is neither transcribed nor fragile in wild-type cells, became fragile following transcriptional activation in a genetically engineered cell line. Previous molecular combing experiments have shown that transcription represses origin firing. Here, we demonstrate that a minimal origin remains partially active under basal transcription conditions, yet is fully inactivated upon transcriptional induction. In contrast, the efficient β-actin promoter/origin retains functionality despite transcriptional activation. The same functional hierarchy emerges in a late-replicating, transcriptionally silent, and non-fragile locus. These results define a class of robust origins that function across diverse chromosomal contexts and support the model in which CFSs arise from transcription-dependent repression of replication origin initiation across large, late-replicating genes. Our study also suggests that depletion of *cis*-elements specifying efficient replication origins contributes to the formation of late-replicating domains, whereas a high density establishes early-replicating regions.

## Graphical Abstract

**Graphical Abstract:**
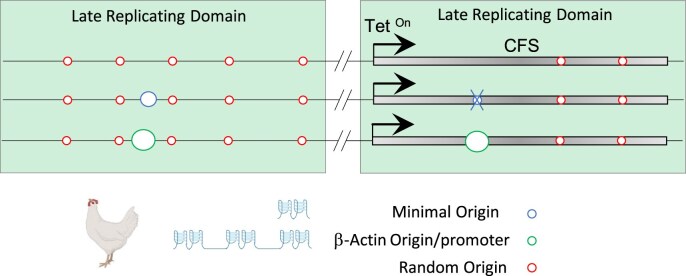

## Introduction

The machinery responsible for replicating the vertebrate genome operates on a template organized into distinct compartments, including early- and late-replicating domains, each posing distinctive challenges [1, 2]. During the licensing period, from late M to the end of G1, two replicative DNA helicases, Mcm2-7, are assembled in their inactive form on chromatin to form the pre-replicative complex (pre-RC) [3, 4]. In the S phase, a highly dynamic process involving a cascade of protein recruitment orchestrated by specific regulatory factors leads to the formation of two CMG helicases (Cdc45.Mcm2-7.GINS) [5, 6]. Their activation leads to DNA unwinding and the initiation of bidirectional replication. Thus, the spatiotemporal pattern of DNA replication results from multiple layers of constraints operating throughout G1 and S phase. The late-replicating compartment is mainly characterized by closed and transcriptionally inactive chromatin, posing significant barriers to both pre-RCs deposition and subsequent activation. Specialized *trans*-acting factors, like the ORC-associated protein, are involved in the facilitation of origin licensing in this closed compartment [7–9]. A key regulator of the replication timing (RT) program is Rif1, which, in association with protein phosphatase 1 (PP1), counteracts DDK-dependent phosphorylation of the MCM double hexamer (MCM-DH), thereby limiting origin activation in late-replicating regions [10–13]. However, the mechanisms regulating the localization and/or activity of Rif1/PP1 in specific domains remain unclear, substantially limiting our understanding of how late-replicating domains are established. Population-based methods failed to identify many initiation sites or zones in late-replicating compartments, suggesting a random pattern of initiation at poorly efficient origins. A well-studied exception to gene paucity of late-replicating domains is the presence of a series of large genes (>300 kb) hosting common fragile sites (CFSs) [14, 15]. CFSs are chromosomal regions that remain incompletely replicated when cells submitted to replication stress enter mitosis [14]. Importantly, CFSs have been shown to be tissue-specific hotspots of chromosome rearrangements associated with cancer progression [16]. Several hypotheses have been proposed to explain how transcription delays the completion of replication at CFSs. One postulates that R-loop formation upon head-on encounter of the replication and the transcription machines blocks replication fork progression and induces fork collapse [17, 18]. However, this model is challenged by evidence showing that copy number variants (CNVs) found in cancer cells cluster within CFSs, but not within regions enriched in highly transcribed genes [18]. Furthermore, depletion of RNase H1, a major contributor to R-loop resolution, does not impact CNVs frequency or distribution. Finally, DRIP-seq analyses showed that CFSs are not enriched in R-loops. Together, these results suggest that R-loops are not the primary driver of transcription-induced CFS instability [19]. A second model relies on the observation that the core of two canonical CFSs is depleted in replication initiation events. This suggests that ongoing transcription actively prevents origin activation [20, 21], leaving these regions more prone to incomplete replication when replication forks slow down. In addition, genome-wide analyses further show that transcription units tend to be depleted in replication initiation events, regardless of RT [22, 23]. This observation suggests a general mechanism by which ongoing transcription would remove the pre-RCs, or prevent further steps in origin assembly and activation. This hypothesis was supported by *in vitro* and *in vivo* studies in *Saccharomyces cerevisiae*, showing that the transcriptional machinery could indeed displace pre-RCs, though this displacement was limited to a few kilobases [24]. *In vivo* experiments, where the well-studied *S. cerevisiae* ARS1 element was artificially inserted into a transcription unit, showed inactivation of the origin [25]. Despite these insights, how transcription modulates the behaviour of efficient vertebrate replication origins across different chromosomal contexts remains poorly explored. However, although sharing many similarities, the nature of the replication machinery (*trans*-factors) and of the replication initiation sites (*cis*-elements) in yeast and vertebrates displays specificities that may lead to different behaviours *in vivo*. Moreover, vertebrate genomes feature more complex chromatin landscapes and regulatory layers, underscoring the need for dedicated *in vivo* studies.

Genome-wide analyses and molecular combing experiments have shown that transcription represses replication origin firing within CFSs. However, key questions remain: can efficient vertebrate origins defined by specific *cis*-determinants, such as G-quadruplex motifs (pG4s), resist this repression? And if so, how are these *cis*-determinants distributed across the human genome? To address these questions, we utilized a well-characterized chicken minimal origin of replication devoid of intrinsic transcriptional activity and containing two pG4s. This origin was inserted into two different late-replicating chromosomal contexts: one embedded within a transcription unit (CFS) and the other within a transcriptionally silent region. We further compared its activity to that of another efficient origin located within the constitutive β-actin promoter, which harbours nine pG4s. Our results demonstrate that transcriptionally active, late-replicating chromatin represses the activity of the minimal origin, with repression levels correlating with transcriptional output. In contrast, the β-actin promoter/origin remains efficient in all tested conditions, suggesting that such elements may be depleted from CFSs and late-replicating domains. Genome-wide analysis of the pG4s density across the human genome reveals marked enrichment in early-replicating regions and depletion in late-replicating regions. This study allows dissection of the molecular interplay between transcription, replication origin activation, and chromatin environment, advancing our understanding of fragile site biology and the establishment of late-replicating domains.

## Materials and methods

### Plasmid construction

The targeting vectors used for homologous recombination in DT40 cells were constructed with the multisite Gateway Pro kit (Thermo Fischer Scientific #12537100). Vectors containing the 5′ and 3′ target arms used for specific recombination at the Late 2 (chr1:177 936 192 bp, galGal5) were described previously [26]. New arms were prepared for specific targeting in the DMD^Tet/Tet^ gene (chr1:114.796.392–114.798.322, galGal5). The 5′ and 3′ target arms for homologous recombination were amplified from DT40 genomic DNA with primer pairs listed in Supplementary Table S1. The arms were oriented so that the minimal origin construct was inserted with the pG4s on the template strand for the active origin version. We used four entry vectors to generate the new minimal origin construct inserted at either the DMD or Late 2 sites: two entry vectors containing the 5′ and 3′ target arms for specific insertion, one entry vector pDONR221 P5-P4 containing the minimal origin described previously [27], and one entry vector pDONR221 P4r-P3r containing the drug selection cassette (β-actin-PuroR at the DMD locus or β-actin-BsR at the Late 2 locus) described previously [28]. Final vectors were generated by recombining compatible *att* sites between the entry vectors, with LR Clonase (Thermo Fisher Scientific #12538120). For electroporation, the final vectors were linearized with *ScaI* (NEB #R3122S).

### Cell culture conditions and transfection

DT40 cells were grown in RPMI 1640 medium supplemented with Glutamax (Thermo Fisher Scientific #61870010), containing 10% foetal bovine serum, 1% chicken serum (Thermo Fisher Scientific #16110-082), 0.1 mM β-mercaptoethanol (Thermo Fisher Scientific #31350010), 200 U/ml penicillin, 200 µg/ml streptomycin, and 1.75 µg/ml amphotericin B at 37°C, under an atmosphere containing 5% CO_2_. Cells were electroporated as previously described. Cell clones were selected on media containing a final concentration of 20 µg/ml blasticidin or 1µg/ml puromycin. Genomic DNA was extracted from cells in lysis buffer [10 mM Tris, pH 8.0; 25 mM NaCl; 1 mM ethylenediaminetetraacetic acid (EDTA) and 200 µg/ml proteinase K]. Clones into which the plasmid DNA was integrated were screened by polymerase chain reaction (PCR) with primer pairs designed to bind on one side of the insertion site, such that one primer bound within the construct and the other primer bound just upstream or downstream from the arm used for recombination (Supplementary Fig. S1 and Supplementary Table S1). The *BsR* or PuroR resistance gene was excised from positive clones using the Cre-LoxP system. DT40 cells constitutively express a tightly regulated Cre recombinase fused to a mutated oestrogen receptor (Mer). This inactive Mer-Cre-Mer fusion protein can be transiently activated in the presence of 4-hydroxytamoxifen, resulting in the efficient excision of genomic regions flanked by two recombination signals (*loxP* sites) inserted in the same direction. For the excision of the genomic DNA flanked by loxP sites, we treated 3 × 10^5^ cells with 5 μM 4-hydroxytamoxifen (Sigma–Aldrich #T176) for 24 h. Subclones were obtained by plating dilutions of the treated cell suspension at a density of 50, 150, and 1500 viable cells per 10 ml in 96-well flat-bottomed microtiter plates. We assessed the excision of the β-actin-BsR or β-actin-PuroR selection cassette by growing cells in the selective media for 72 h, with a control plate without selection. Cell clones that died in the presence of the selection drug were selected for the correct excision. For each clonal line, the copy number of the construct was quantified by quantitative PCR (qPCR) with one primer pair amplifying inside the transgene (with) and another one amplifying in the mid-late locus on both alleles (both) (Supplementary Table S2). Transcriptional activation of the DMD^Tet/Tet^ gene was achieved by treating cells with 1 µg/ml tetracycline for 24 h.

### SNS purification

Small nascent strand (SNS) purification was performed as previously described with some slight modifications. Fresh or frozen cultured cells were used for total genomic DNA extraction and the T4 polynucleotide kinase (Biolabs #M0201S) concentration was adjusted to 100 U and incubated for 30 min at 37°C. Proteinase K (Thermo Fischer Scientific #EO0491) digestion was realized at a final concentration of 625 µg/ml for 30 min at 50°C. We used 500 U of a custom-made λ-exonuclease (Thermo Fischer Scientific #EN056B1C002, 50 U/ml) for each sample preparation.

### Replication timing analysis

For RT experiments, ~10^7^ exponentially growing cells were pulse-labelled with 5-bromo-2′-deoxyuridine (BrdU, Sigma–Aldrich #B9285) for 1 h and sorted into four S-phase fractions, from early to late S phase. The collected cells were treated with lysis buffer (50 mM Tris, pH 8.0; 10 mM EDTA, pH 8.0; 300 mM NaCl; 0.5% sodium dodecyl sulphate (SDS); 0.2 mg/ml of freshly added proteinase; and 0.5 mg/ml of freshly added RNase A), incubated at 56°C for 2 h, and stored at –20°C in the dark. Genomic DNA was isolated from each sample by phenol–chloroform extraction and alcohol precipitation and sonicated four times for 30 s each, at 30 s intervals, in the high mode at 4°C in a Bioruptor water bath sonicator (Diagenode), to obtain fragments of 500–1000 bp in size. The sonicated DNA was denatured by incubation at 95°C for 5 min. We added monoclonal anti-BrdU antibody (BD Biosciences #347580) at a final concentration of 3.6 μg/ml in 1× IP buffer (10 mM Tris, pH 8.0; 1 mM EDTA, pH 8.0; 150 mM NaCl; 0.5% Triton X-100; and 7 mM NaOH). We used 50 µl of protein-G-coated magnetic beads (Thermo Fisher Scientific #10004D) per sample to pull down the anti-BrdU antibody. Beads and BrdU-labelled nascent DNA were incubated for 2–3 h at 4°C on a rotating wheel. The beads were then washed once with 1× IP buffer, twice with wash buffer (20 mM Tris, pH 8.0; 2 mM EDTA, pH 8.0; 250 mM NaCl; 0.25% Triton X-100), and then twice with 1× TE buffer, pH 8.0. The DNA was eluted by incubating the beads at 37°C for 2 h in 250 µl 1× TE buffer pH 8.0, to which we added 1% SDS and 0.5 mg/ml proteinase K. DNA was purified by phenol-chloroform extraction and alcohol precipitation and resuspended in 50 µl TE. The BrdU-labelled nascent strands (NSs) quantification was performed as previously described [28–30].

### Flow cytometry analysis and sorting

After BrdU incorporation, DT40 cells were washed twice with phosphate buffered saline (PBS), fixed in 75% ethanol, and stored at −20°C. On the day of sorting, fixed cells were resuspended at a final concentration of 2.5 × 10^6^ cells/ml in 2% foetal bovine serum in PBS (Sigma, #CA-630), 50 μg/ml propidium iodide, and 0.5 mg/ml RNase A, and incubated for 30 min at room temperature. Singlet cells were sorted with a FACSAria Fusion (BD Biosciences). Four fractions of S-phase cells (S1–S4), each containing 5 × 10^4^ cells, were collected and further treated for locus-specific RT analyses.

### Extraction of 5-ethynyl uridine incorporated nascent RNAs and reverse transcription

Exponentially growing DT40 cells were treated with 0.5 mM 5-ethynyl uridine for 15 min or 1 h. Transcriptional activation of the DMD^Tet/Tet^ gene, when required, was achieved by treating cells with 1 µg/ml tetracycline for 24 h before EU labelling. 5-ethynyl uridine-labelled RNA was captured using the Click-iT Nascent RNA Capture Kit (Thermo Fischer Scientific, #C10365) according to the manufacturer’s instructions. Total RNA was extracted from 10 × 10^6^ cells with the miRNeasy kit (Qiagen, #217004). Five micrograms of total RNA was biotinylated thanks to the click reaction between 5-ethynyl uridine and azide-modified biotin. Two micrograms of biotinylated RNA was then purified using streptavidin magnetic beads. Nascent RNAs were reverse transcribed on the beads (RT^+^) using Superscript VILO complementary DNA (cDNA) Synthesis Kit (Thermo Fischer Scientific, #11754050). Negative controls (RT^−^) were performed with the same procedure but without the addition of reverse transcriptase. The comparison of RT^+^ and RT^−^ samples was used to validate the absence of genomic DNA in the RNA samples. Real-time qPCR was performed using primer pairs listed in Supplementary Table S1. RNA levels were calculated relative to the Bu1a expression level. Nascent RNA quantification experiments were performed using two clonal cell lines per analysed construct.

### Real-time PCR quantification of DNA

Real-time qPCRs were executed according to the Minimal Information for Publication of Quantitative Real-Time PCR Experiments (MIQE) guideline. The LightCycler 480 Real-time PCR system with the SYBR Green I Master Mix (Roche Life Science, #04887352001) was used for the real-time PCR quantification of BrdU-labelled NSs, genomic DNA extracted from clonal cell lines, short NSs, or 5-ethynyl uridine incorporated nascent RNAs. Each sample was quantified at least in duplicate. For all reactions real-time PCR was performed under the following cycling conditions: initial denaturation at 95°C for 5 min, followed by 50 cycles of 95°C for 10 s, 61°C for 20 s, 72°C for 20 s, and fluorescence measurement. Following amplification, a thermal melting profile was used for specific amplicon validation. Raw Cq values were calculated using the LightCycler 480 SW 1.5.0 software using the second derivative Max type for analysis, according to RDML guidelines (http://www.rdml.org).

### Sequencing library preparation

Sequencing libraries were prepared using NEBNext Ultra II Directional RNA Library Prep Kit for Illumina (NEB #E7760S), following the manufacturer’s instructions for RT-nascent RNAs. The samples were not subjected to size selection, but were cleaned up for adaptor-ligated DNA using a SPRISelect Reagent Kit (Beckman coulter #B23317). Libraries were prepared from cDNA produced using 1 µg of biotinylated EU-RNA as a starting amount. A 10-fold diluted adaptor was used for the adaptor ligation. Library amplification was performed using the Unique Dual Index UMI Adaptors RNA Set 1 (NEB#E7416) with 12 PCR cycles. The mean size and quality of the library molecules were determined using an Agilent Bioanalyser High Sensitivity DNA chip (Agilent Technologies, #5067–4626).

### Sequencing and data processing

Sequencing was performed by Novogene using an Illumina NovaSeq X Plus sequencer with 10 B flow Cell. Samples were sequenced in paired-end mode (150 bp paired-end reads) according to standard procedures. Approximately 10 M of reads (∼3 Gb) were generated per sample. Paired-end reads were aligned to the Gallus gallus genome assembly galGal5 using bowtie2 (version 2.5.1) with default parameters. Resulting SAM files were processed using Samtools (version 1.21). BAM files were indexed with samtools index. Processed BAM files were converted into BigWig coverage files using the Galaxy France platform for visualization in the UCSC Genome Browser (https://genome.ucsc.edu/). Coverage tracks were generated by applying a mean windowing function with a smoothing window size of 2 pixels. The SNS coverage profiles at the β-actin locus on chromosome 14 were obtained from a previous analysis [31].

### Databases

The genome assembly hg19 was downloaded from UCSC to calculate the GC% for every bin of 50 kb genome-wide. The pG4 detections, which correspond to automatic detections combining pattern matching and manual curation, were downloaded from Zheng *et al.*, 2020 [32]. Clustered pG4s were detected using Python (v3.12.3) and pandas (v2.3.3) with a maximum distance between pG4s allowed for pG4s to be merged of 100 bp on the same strand. Clusters of pG4s from both strands were combined to generate the circos plots, violin plots, and transcription start site (TSS) profiles. pG4 cluster coverage was quantified in 50 kb bins for both hg19 and hg38 assemblies using pyranges (v0.1.4). RT data for JEFF lymphoblastoid cells were obtained from Repli-seq S1–S6 phases (Brison *et al.*, 2019 [33]; GEO : GSE134709). To determine the three classes of genes used in Fig. 6, 116 genes that were annotated in GENCODE v7 (hg19) were extracted from Significantly Delayed Region/Significantly Delayed Window (SDR/SDW, Brison *et al.*, 2019), of which 84 are >200 kb and are annotated as CFSs. The second class comprises long non-CFS and corresponds to the remaining genes >200 kb (*n* = 1 584). The third class corresponds to genes <200 kb that do not overlapping an SDR/SDW (*n* = 49 210). pG4 coordinates were converted to hg38 assembly using the LiftOver tool from UCSC. The genome annotation Ensembl 110 (GRCh38) was used to generate violin plots and TSS profiles.

### Replication timing segmentation

BigWig files obtained from Brison *et al.*, 2019 were converted to bedGraph using the binary file bigWigToBedGraph with default parameters downloaded from UCSC, and RT scores computed in non-overlapping 50 kb bins using the ENCODE six-phase formula (Du, Q *et al.*, 2019 [34]): ​RT = (S1 × 0.917) + (S2 × 0.75) + (S3 × 0.583) + (S4 × 0.417) + (S5 × 0.25) + (S6 × 0). Scores were normalized by min–max scaling (0 = latest, 1 = earliest). After exclusion of ENCODE hg19 blacklist regions (centromeres, difficult-to-sequence loci), the mappable genome was ranked by RT and partitioned into five quintiles (20% each): Early, Mid-early, Mid, Mid-late, and Late.

### Circos plots

Chromosome 1–5 circos plots were generated with R (v4.3.2) in hg19 assembly. dplyr (v1.1.4) was used for efficient data filtering and chromosome-specific partitioning. GenomicRanges (v1.54.1) enabled the masking of blacklist regions from the analysis, while visual bridging was used to maintain track continuity across these masked areas using the circlize package (v0.4.16). All profiles were generated using 50 kb bins and 21-bin sliding window smoothing. Tracks: pG4 clusters coverage (fraction per bin, fourth-root transformed: *y* = *x*^0.25^); RT (min–max scaled 0–1); GC content (% GC per bin, min–max scaled 0–1, 0 = lowest, 1 = highest); CFSs. Top 20% earliest replicating (grey) and the latest replicating (orange) regions were highlighted.

### Violin plots

Log_2_-transformed pG4 clusters coverage was computed across entire gene bodies or gene bodies excluding the first 2 kb downstream of the TSS to mitigate TSS bias. Statistical significance was assessed by the non-parametric Kruskal–Wallis test (****P < .0001). Violin plots were generated using GraphPad Prism 10.

### TSS profile

The fraction of pG4 cluster coverage was calculated at base-pair resolution around TSS (±10 kb) and smoothed with a 200 bp sliding window. Profiles are shown for CFS genes, long non-CFS genes >200 kb, and genes <200 kb. The plots were generated in Python (v3.12.3) using numpy (v1.26.4), Matplotlib (3.10.7) and intervaltree (v3.1.0) packages.

## Results

### Ongoing transcription across the late-replicating DMD gene suppresses firing of the β-globin minimal origin

**Figure 1.**
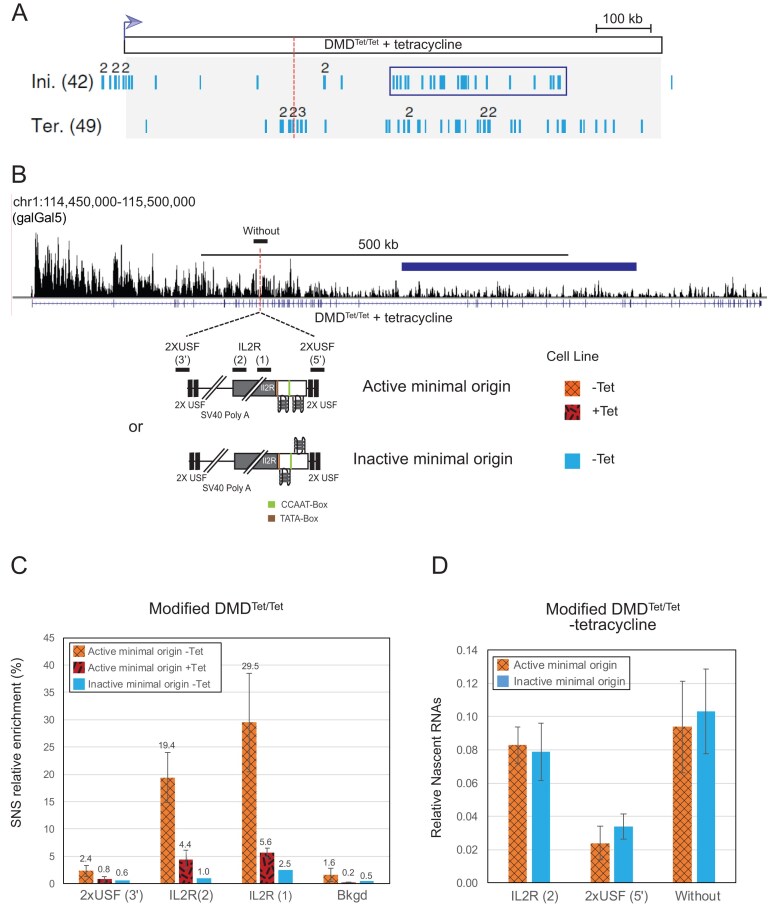
Transcription through the DMD^Tet/Tet^ fragile site represses origin firing. (**A**) Schematic representation of replication initiation (Ini) and termination (Ter) events identified by molecular combing in DMD^Tet/Tet^ cells grown in the presence of tetracycline (Blin *et al.*, 2019). The dotted line indicates the insertion site for the active or inactive minimal origin. The rectangle surrounds a zone of initiation, which is also represented in panel (B). (**B**) UCSC Genome Browser view showing EU-seq signal across the DMD gene in the DMD^Tet/Tet^ cells grown in the presence of tetracycline. Although the studied cell line is devoid of insertion, the insertion site for the active or inactive minimal origin is indicated by a dotted line. Below are schematic representations of the inserted constructs: each construct is flanked by two USF (Upstream Stimulating Factor) binding sites and contains a 90 bp minimal origin composed of two pG4s on the same strand (dimeric pG4). The second one is surrounded by a CCAAT and a TATA box, represented in green and brown, respectively. In the active origin, the G-tracks are on the template strand. The inactive minimal origin is similar to the active one, except that the first pG4 is on the reverse-complement strand. Lines above the construct indicate the positions of amplicons used for SNS and nascent RNA quantifications, specific to the modified chromosome. The amplicon above the DMD gene overlaps the insertion site and is specific to the unmodified chromosome (without). (**C**) SNS relative enrichments along the ectopic active minimal origin obtained in clones (*n *= 2, biological replicates) cultured without (black diagonal hatching pattern) and with (random dash pattern) tetracycline, or along the inactive minimal origin in clones (*n *= 1) cultured without tetracycline (plain pattern). The mean values are indicated above each bar. Error bars correspond to standard deviation (SD). The amplicons used for quantification are indicated in panel (B). Bkgd refers to an amplicon located 5 kb away from the insertion site. An amplicon within the endogenous ρ-origin was arbitrarily set at 100% to calculate the SNS relative enrichment. (**D**) Relative abundance of nascent RNAs at and around the minimal origin. Nascent transcripts were measured in two cell lines (*n *= 2, biological replicates) containing the active (black diagonal hatching pattern) or the inactive (plain pattern) minimal origin within the DMD^Tet/Tet^ cell line, grown without tetracycline. Error bars correspond to SD. Nascent transcripts levels at the endogenous Bu1a gene were arbitrarily set at 1 for normalization. The amplicons used for RT-qPCR quantification are shown in panel (B).

To determine whether ongoing transcription within large genes can suppress replication origin firing in DT40 cells, we analysed the behaviour of the β^A^-globin minimal origin inserted into an appropriately engineered DMD gene. Indeed, we took advantage of a cell line in which both alleles of this late-replicating and 1 Mb long gene, which is naturally silent and non-fragile in wild-type (WT) DT40 cells, have been replaced by the tetracycline-inducible promoter (DMD^Tet/Tet^) [21]. In the absence of tetracycline treatment, these cells exhibit low levels of transcription and gene fragility, two features that were accentuated upon tetracycline treatment. A previous molecular combing analysis has revealed that in WT cells, initiation sites are randomly distributed across the gene. However, in tetracycline-treated DMD^Tet/Tet^ cells, initiation sites are excluded from a region extending over ~500 kb within the 5′ part of the gene, resulting in a large termination zone (Fig. 1A). Despite transcriptional activation, the core of the gene remained late-replicating, and the termination zone was shown to host the fragile region [21]. Given that replication initiation exclusion is limited to the first half of the DMD gene, we wondered if the transcription rate was similar in the 5′ and 3′ regions of this long gene. To address this, we labelled asynchronous DMD^Tet/Tet^ cells with EU for 15 min. After the capture of newly synthesized RNA and reverse transcription, the nascent transcript levels were analysed by EU-seq. Notably, we observed a marked decrease in the number of nascent transcripts in the second half of the gene, which corresponds to the region where initiation events are preserved (Fig. 1B).

These findings support the model positing that transcription-induced remodelling of replication origin distribution prevents replication completion of large genes before mitosis upon fork slowing, resulting in fragility. To directly assess the impact of passage of the transcription machinery on origin activity in a controlled setting, we employed a previously characterized efficient β^A^-globin minimal origin devoid of promoter activity [27]. This 90 bp minimal origin, which contains two potential G-quadruplexes motifs on the same DNA strand (dimeric pG4, Fig. 1B), was inserted by homologous recombination into the centre of the DMD termination zone on one chromosome in DMD^Tet/Tet^ cells (Fig. 1B). Given that ~30% of efficient origins in human and chicken cells share this organization, the minimal origin serves as a representative model for efficient replication initiation sites. To prevent premature transcription termination caused by the SV40 poly(A) signal in the construct, the active minimal origin was inserted with G-tracks aligned to the template strand (Fig. 1B). Quantification of the SNS relative enrichment revealed that in the absence of tetracycline induction, the minimal origin remains functional, albeit its efficiency decreases significantly (ninefold) when compared with previously tested insertion in mid-late-replicating region (Fig. 1C and Supplementary Table S3A). Supporting a model in which transcription suppresses initiation events, we observed an additional sixfold decrease in initiation efficiency upon tetracycline addition (Fig. 1C and Supplementary Table S3A). To confirm the specificity of the SNS relative enrichment signal detected in the absence of tetracycline induction, we analysed a control cell line containing an inactive version of the minimal origin at the same locus (Fig. 1B) [27]. In this control, SNS relative enrichment was tenfold lower than with the active minimal origin (Fig. 1C and Supplementary Table S3A). Overall, these results strongly support the hypothesis that transcription inhibits the initiation at an efficient origin.

### Transcription progresses unimpeded through an active origin in a late-replicating region

To determine whether the presence of an efficient origin affects RNA polymerase II progression, we quantified the amount of nascent transcripts immediately downstream of the minimal origin (Fig. 1D and Supplementary Table S3B). We used an amplicon overlapping the insertion site to quantify nascent transcripts on the unmodified chromosome (amplicon without, Fig. 1B). We compared this expression with that observed on the modified chromosome both upstream and downstream of the minimal origin [IL2R (2) and 2XUSF (5′) respectively, Fig. 1B]. As an additional control, we also analysed two cell lines in which the inactive minimal origin was inserted at the same site. As a reference, we quantified the transcripts from the *Bu1a* gene, arbitrarily setting its expression level to one. To ensure sufficient cDNA yields for qPCR analysis, nascent RNAs were labelled with EU for 1 h as previously described [21]. Thus, the measured RNA levels reflect both the transcriptional activity and the stability of the analysed transcripts. To minimize the impact of the construct insertion on RNA synthesis, we introduced the construct within an intron. We observed a two-fold decrease in nascent transcript levels downstream of the minimal origin (Fig. 1D and Supplementary Table S3B). This reduction was consistent for both active and inactive origins, suggesting that the presence of pG4 structures may impede RNA polymerase II progression in this specific chromosomal context. RT-qPCR analysis of nascent RNAs revealed that, although the signal after the origin was reduced, it remained clearly above background, as no amplification was observed in the negative control lacking reverse transcriptase (Supplementary Table S3B). This suggests that RNA polymerase progression through the minimal origin occurs. To compare the transcriptional activity of the DMD gene in its uninduced and induced states, we analysed nascent transcript levels, labelled for 15 min with EU, across the entire gene by EU-seq. We observed an ~2.5-fold increase in transcription between the −Tet and +Tet conditions, consistent with our RT-qPCR results and previously published data (Supplementary Fig. S2A) [21]. Overall, our results support the model proposing that transcriptional progression through the long DMD gene inhibits origin firing, whereas firing of an intragenic origin does not affect transcription of this large, late-replicating gene.

### The β-actin origin/promoter remains efficient when inserted into the DMD^Tet/Tet^ gene

**Figure 2.**
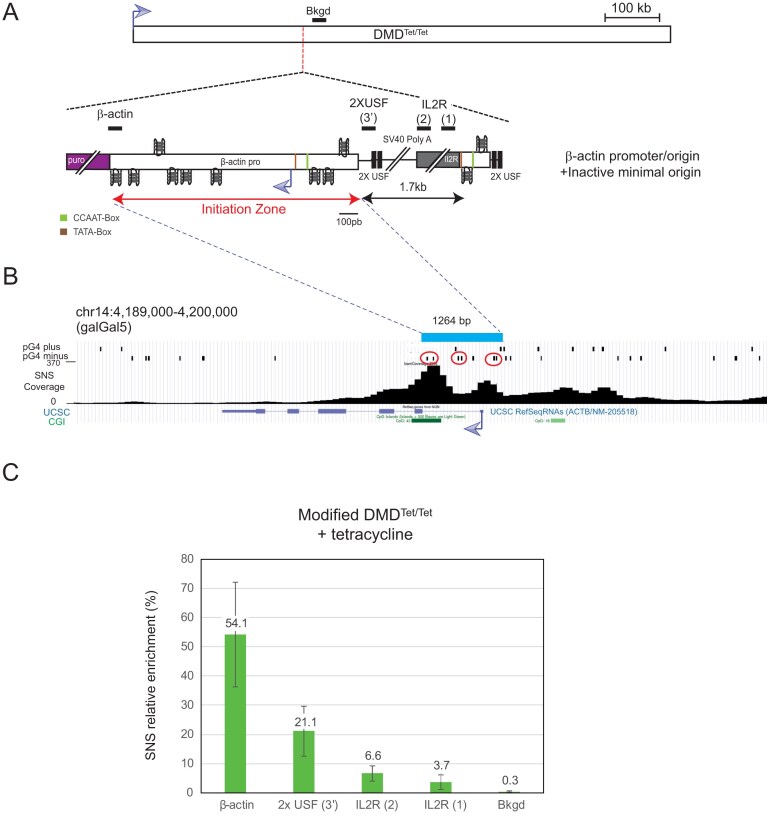
The β-actin promoter/origin is not repressed when inserted within the transcribed DMD gene. (**A**) Schematic representation of the DMD^Tet/Tet^ cell line containing the β-actin promoter/origin fused to the minimal inactive origin. Lines indicate the position of amplicons used for SNS quantifications, specific to the modified chromosome. The dotted line indicates the site of insertion. (**B**) The endogenous β-actin promoter is located within a replication initiation zone. The top bar defines the 1264 bp region derived from the β-actin promoter present in the construct. Below, pG4 detected on the plus and minus strands in Zheng *et al.*, 2020 [32] are shown. Dimeric pG4s found within the ectopic β-actin promoter are surrounded by ellipses. CpG islands (CGI) and UCSC RefSeq RNA annotations are also indicated at the bottom. (**C**) SNS relative enrichments along the ectopic active β-actin promoter/origin and the minimal inactive origin obtained in clones (*n* = 2, biological replicates) cultured with tetracycline for 24 h. The mean values are indicated above each bar. Error bars correspond to SD. The amplicons used for quantification are indicated in panel (A). Bkgd refers to an amplicon located 5 kb away from the insertion site. An amplicon within the endogenous ρ-origin was arbitrarily set at 100% to calculate the SNS relative enrichment.

To further investigate how transcription affects origin firing within the DMD gene, we analysed cell lines containing the minimal inactive origin before excision of the strong β-actin promoter/origin, which drives the selection gene expression (Fig. 2A). This β-actin promoter contains nine pG4s (six of which are within dimeric pG4s). Consistent with the model proposing that dimeric pG4s are efficient replication initiation sites, SNS-seq analysis in WT DT40 cells revealed a pronounced enrichment zone covering the endogenous β-actin promoter (Fig. 2B). Previous work demonstrated that a 1264 bp fragment encompassing this promoter induces efficient replication initiation when ectopically inserted in a mid-late-replicating region [30]. Accurately assessing the global activity of this ectopic origin by SNS relative enrichment is challenging due to the presence of the endogenous β-actin locus. To circumvent this limitation, we quantified SNS relative enrichment using an amplicon spanning the junction between the β-actin promoter and the puromycin resistance gene, located within the initiation zone detected at the endogenous locus (Fig. 2A and B). We observed significant enrichment at this junction, which was further supported by enrichment detected at the 2 × USF (3′) amplicon located at the other side of the initiation zone (Fig. 2C and Supplementary Table S4A). In contrast, the weak enrichment observed at IL2R (1) confirmed the absence of origin activity at the minimal inactive origin. Because SNS preparations were performed in cells cultured in the presence of tetracycline, these results indicate that the β-actin promoter/origin remains functional even within the highly repressive context of a CFS.

**Figure 3.**
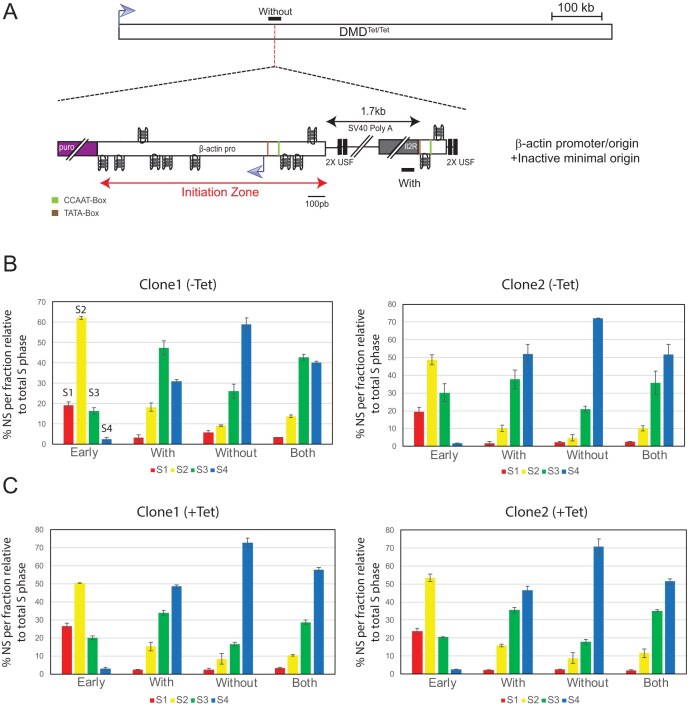
The β-actin promoter/origin locally advances the RT of the transcribed DMD gene. (**A**) Schematic representation of the DMD^Tet/Tet^ cell line containing the β-actin promoter/origin fused to the minimal inactive origin. Lines indicate the position of amplicons used for allele-specific quantifications of RT profiles. The dotted line indicates the site of insertion. (**B**) RT profiles of each chromosomal allele were determined after targeted transgene integration, using the allele-specific analysis by qPCR. BrdU pulse-labelled cells were sorted into four S-phase fractions, from early to late (S1–S4), and the immunoprecipitated newly synthesized strands (NS) were quantified by qPCR in each fraction. Error bars correspond to SD from qPCR replicates. Specific primer pairs determine the RT profile for the modified allele (with), the WT allele (without), and 5 kb away from the insertion site on both alleles (both). The endogenous β-globin locus was analysed as an early-replicated control (early). Two independent clones containing the indicated constructs were analysed. (**C**) Same experimental procedure as in panel (B), except that the cells were grown for 24 h in the presence of tetracycline before RT analysis.

To validate this observation, we next assessed the impact of this origin on RT, as a shift in RT can reflect origin efficiency, as previously observed [27–30]. RT shift analysis of two independent cell lines indicated that the β-actin origin alone is sufficient to induce a shift towards earlier replication when cells were grown without tetracycline, although the region remains replicated in the second half of S phase (Fig. 3B and Supplementary Table S4B). This contrasts with the incapacity of the minimal origin to shift the RT under similar conditions but aligns with the low SNS relative enrichment observed at the minimal origin (Supplementary Fig. S2B, Supplementary Table S4D, and Fig. 1C). To further explore the capacity of the β-actin promoter/origin to remain efficient when transcription is up-regulated, we also test its capacity to advance RT upon tetracycline induction. We observed no significant difference in RT shift with or without tetracycline, suggesting that the β-actin origin maintains its firing capacity despite transcription induction (Fig. 3C and Supplementary Table S4C). This finding is consistent with the significant SNS enrichment detected around the ectopic origin in tetracycline-treated cells (Fig. 2C).

These results show that the efficiency of the origins of replication depends largely on the chromosomal context in which they are found. While the minimal origin and the β-actin origin exhibit similar efficiencies, based on RT shifts, in a region replicated in the mid-late S phase [30], the β-actin origin is more efficient within a late replication region and is also able to remain functional when crossed by RNA polymerase II. On the other hand, the activity of the minimal origin is greatly affected in a late-replicating context, with an increase in the transcription rate leading to its complete inhibition.

### Replication origin efficiency hierarchy is conserved in a silent, late-replicating domain

**Figure 4.**
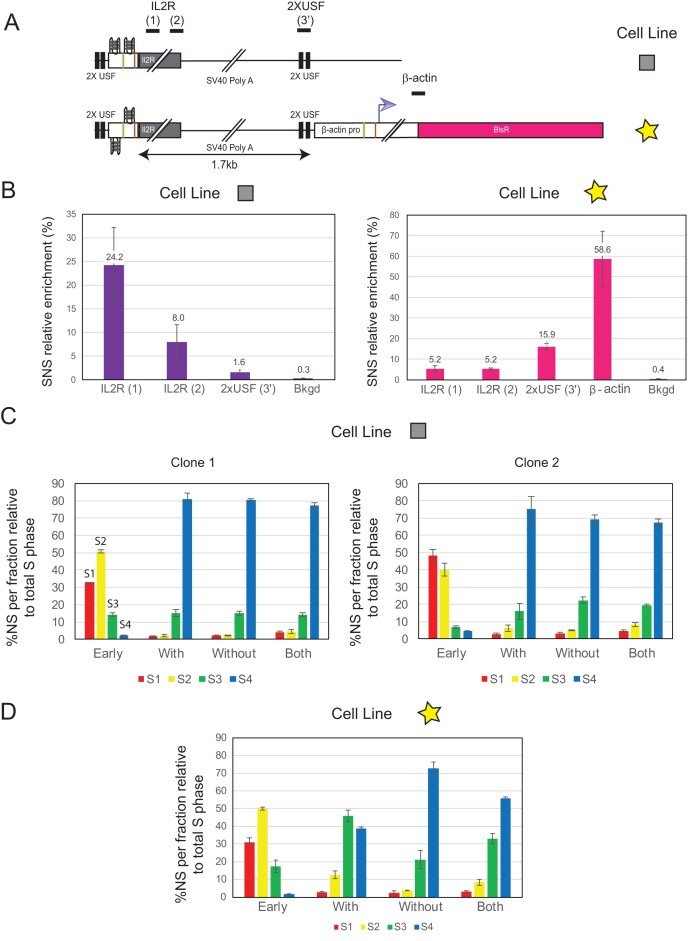
A late chromosomal environment represses the minimal efficient origin but not the β-actin promoter/origin. (**A**) Schematic representation of the two analysed cell lines. Both constructs were inserted at the Late2 locus (chr1:177,936,192 bp, galGal5) previously described in Duriez *et al.*, 2019, and Brossas *et al.*, 2020. One cell line harbours the active minimal origin alone (square), while the other contains the inactive minimal origin fused to the β-actin promoter/origin (star). Lines above the construct indicate the position of amplicons used for SNS quantifications. (**B**) SNS relative enrichments along the ectopic active or inactive minimal origin and the β-actin promoter/origin obtained in clones (*n* = 2, biological replicates), the mean values are indicated above each bar. Error bars correspond to SD. The amplicons used for quantification are indicated below each graph. Bkgd refers to the amplicon located 11 kb away from the integration site. One amplicon within the endogenous ρ-origin was arbitrarily set at 100% to quantify the relative SNS abundance. (**C**) RT profiles of each chromosomal allele were determined after targeted transgene integration, using the allele-specific analysis by qPCR. Specific primer pairs determine the RT profile for the modified allele [with, amplicon IL2R (1)], the WT allele (without, amplicon overlapping the site of insertion), and 3.5 kb away from the insertion site on both alleles (both). The endogenous β-globin locus was analysed as an early-replicated control (early). Analyses of two independent clones containing the active minimal origin inserted at the Late2 locus. Error bars correspond to SD from qPCR replicates. (**D**) RT profiles for one clone containing the minimal inactive origin next to the β-actin promoter inserted at the Late2 locus are shown.

We tested the effect of transcription passing through a minimal efficient origin and the β-actin promoter/origin in a late-replicating CFS. We observed different behaviours, demonstrating that the chromosomal context imposes specific constraints on the replication machinery. In order to test the impact of a late replication context without transcription, we initially tried to insert the minimal origin in the WT DMD context. Despite numerous attempts, we were unable to obtain clones that had inserted the construct by homologous recombination, likely due to the poor chromatin accessibility in this silent region, which impairs efficient homologous recombination. As an alternative, we tested a late-replicating region for which we had already produced several clones, showing that homologous recombination is feasible in this silent and late-replicating region. The timing of replication is comparable to that found in the DMD gene, and the locus was shown to be tightly bound to the nuclear periphery and to have a tight late-replicating control [26]. In this late-replicating region, SNS relative enrichment analysis showed that the minimal origin was repressed compared to the mid-late-replicating domain, but exhibited a level of SNS relative enrichment similar to that observed when inserted in the DMD gene of the DMD^Tet/Tet^ cell line and in the absence of tetracycline induction (Fig. 4B and Supplementary Table S5A, graph on the left compared with Fig. 1C, orange bars). This result aligns with the absence of any RT shift towards earlier replication of the modified chromosome (Fig. 4C and Supplementary Table S5B). As a control, we quantified SNS relative enrichment in a cell line containing the inactive minimal origin adjacent to the active β-actin promoter/origin. SNS relative enrichment at the inactive minimal origin was approximately fivefold lower than at the active minimal origin (Fig. 4B and Supplementary Table S5A, on the right, enrichment at IL2R (1 and 2) is fivefold lower), confirming the weak but detectable activity of the active minimal origin at this locus. However, we observed a more pronounced SNS relative enrichment at the ectopic β-actin origin, similar to that observed when inserted in the DMD gene of the DMD^Tet/Tet^ cell line and in the presence of tetracycline induction. RT shift analysis of this cell line indicated that the β-actin origin is sufficient to induce a shift towards earlier replication, although the region remains late-replicating (Fig. 4D and Supplementary Table S5C). The extent of this shift is comparable to that seen in the transcribed DMD gene, suggesting that the β-actin origin is active in a substantial fraction of cells and functions as an efficient origin in this repressive context. RT shift analysis of clones containing the β-actin origin associated with the active minimal origin confirmed the capacity of the β-actin origin to significantly advance the RT of this late locus (Supplementary Fig. S3 and Supplementary Table S6). Overall, this chromosomal context behaves similarly to the uninduced DMD^Tet/Tet^ gene, exerting a global repressive effect on origin activity of the minimal origin, although the β-actin promoter/origin retains its capacity to advance RT. This result underscores the significant influence of the chromosomal environment on origin function. It also suggests that a strong promoter, associated with efficient replication origins containing multiple dimeric pG4 motifs, establishes a chromatin context around the promoter that is highly conducive to origin activation.

### Long genes and late-replicating domains are depleted in pG4 clusters

**Figure 5.**
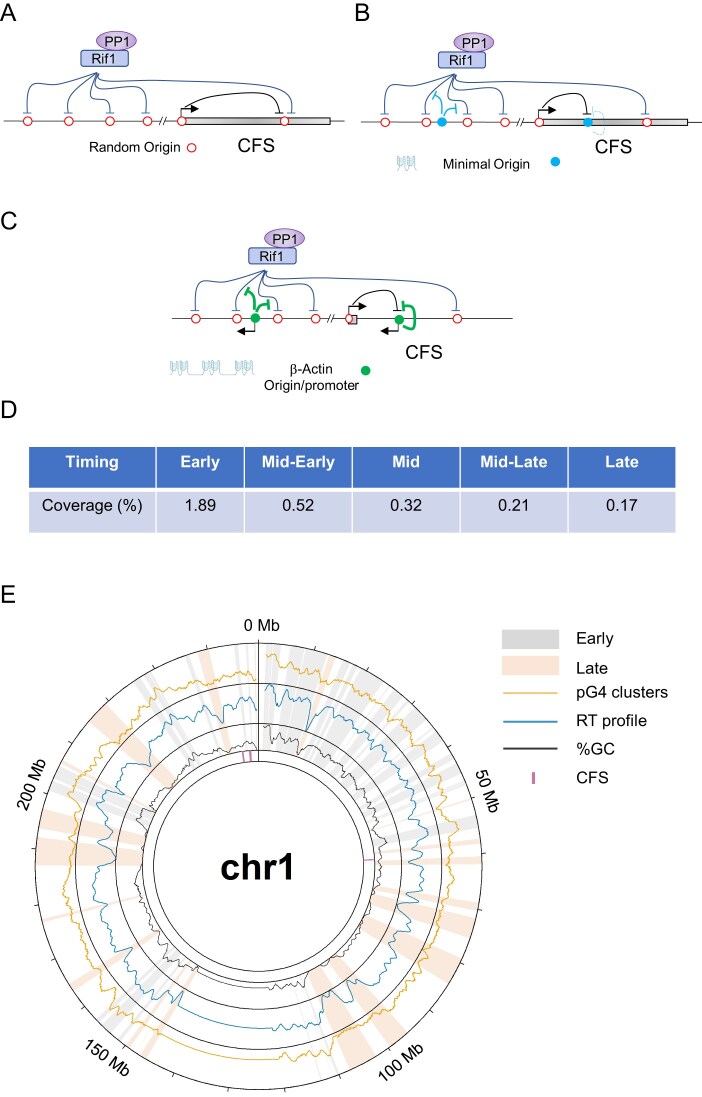
Late chromosomal environments are depleted in *cis*-elements defining efficient replication origins. (**A**) Schematic representation of the repressive role of Rif1 and transcription on replication origin activity along a late-replicating domain and a CFS. (**B**) The minimal origin can counteract global repression operating within a late-replicating domain. However, increasing the level of transcription along a CFS fully represses its activity. (**C**) The β-actin origin/promoter is efficient in the two repressive contexts. (**D**) Coverage of pG4 clusters across five equal sections of the human genome ranging from early to late. (**E**) Circos plot showing from the outside in pG4 clusters coverage, RT profile, GC%, and CFSs across human chromosome 1 (hg19). The top 20% earliest and latest replicating regions are highlighted in grey and orange, respectively.

We recently reported that 30% of highly efficient human replication origins contain pG4 dimers (two pG4 motifs located on the same strand and separated by no >100 bp) and that 50% of pG4 dimers contain an origin detected by SNS-seq [27]. We showed that the addition of the β-actin promoter/origin, containing three pG4 dimers, is sufficient to advance the timing of replication locally of two late-replicating loci. Although the timing remains late, the significant shift towards earlier replication indicates the formation of an efficient initiation site or zone. Potential replication origins located within late-replicating domains are negatively regulated by Rif1, which, in association with PP1, counteracts DDK-dependent phosphorylation of the MCM-DH, thereby limiting origin activation [10–13]. We hypothesized that the two very late-replicating regions investigated in this study are also under the repressive control of Rif1/PP1 (Fig. 5A). If this is the case, the insertion of the β-actin promoter—and, to a lesser extent, the minimal origin—is sufficient to locally counteract Rif1/PP1 activity. This is evidenced by the detection of localized replication initiation events through SNS enrichment and a shift in RT at the β-actin origin (Fig. 5B and C). Moreover, we previously showed that the β-actin promoter/origin, inserted at two sites separated by 30 kb in a region replicated in mid-late S phase, ensured the formation of an early-replicating domain [30]. Taken together, these data suggest a model in which late replication domains form due to a lack of *cis*-regulatory elements ensuring efficient replication initiation. In accordance with this model, the depletion of *cis*-elements that bind transcription factors causes the shift of an early domain to a late domain in mouse embryonic stem cells [35].

To test whether pG4 dimers are depleted in late-replicating domains compared with early-replicating domains, we analysed the genome-wide coverage of pG4 dimers across the human genome. Since in many cases the number of grouped pG4s exceeds two, we will refer to them as a pG4 cluster. To analyse the distribution of *cis*-elements capable of inducing efficient origin formation, we measured the coverage of pG4 clusters in regions with distinct RT. We chose RT data obtained from the lymphoblastoid JEFF cell line because CFSs were also mapped across the entire genome in this cell line [33]. The genome was divided into five equal sections, each corresponding to a different RT, ranging from early to late. Analysis of pG4 clusters coverage shows a 10-fold enrichment of these elements in early regions compared to late regions suggesting a key role of this element in defining early and late-replicating compartments (Figs. 5D-E and Supplementary Fig. S4).

**Figure 6.**
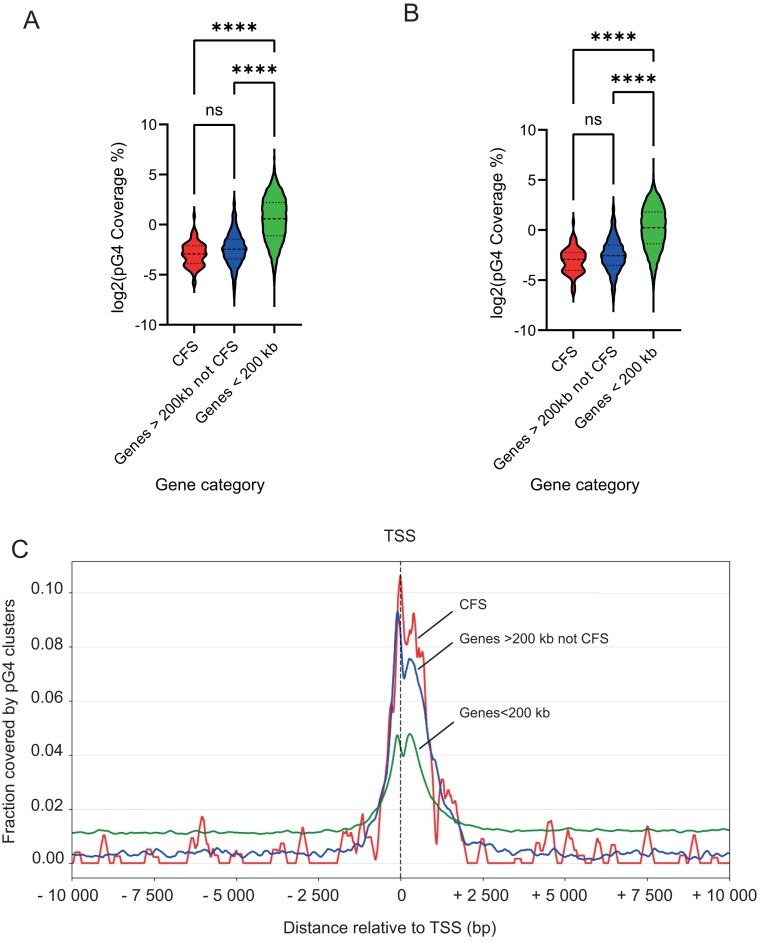
pG4 clusters are depleted from long genes but enriched at their TSS compared to smaller genes. (**A**) Coverage of pG4 clusters along CFSs mapped in lymphoblasts (Brison *et al.*, 2019) (*n *= 84), genes >200 kb that are not CFSs (*n *= 1584), and genes <200 kb (*n *= 49 210). A non-parametric one-way ANOVA (Kruskal–Wallis-test) was used for statistical analysis. ****P-value < .0001. (**B**) Same plot as panel (A), except that the first 2 kb downstream of the TSS were excluded. (**C**) Coverage of pG4 clusters around the TSS of CFSs mapped in lymphoblasts (Brison *et al.*, 2019), genes >200 kb that are not CFSs and genes <200 kb.

To assess the likelihood of detecting this *cis*-element within CFSs, we examined the coverage of pG4 clusters within coding regions. We used CFSs mapped genome-wide in lymphoblasts [33]. We defined three gene classes: (i) genes identified as CFSs in human lymphoblasts; (ii) long genes (>200 kb) that are not classified as CFSs in lymphoblasts; and (iii) genes shorter than 200 kb. We found that the genomic coverage of pG4 clusters is significantly reduced in long genes, irrespective of their fragile status (Fig. 6A).

Analysis of pG4 clusters coverage within a 10 kb window surrounding TSSs revealed enrichment at TSSs, consistent with previous observations for pG4 motifs in general [36]. Notably, long genes exhibited a higher density of pG4 clusters at their TSSs compared with short genes, a trend that was further accentuated at TSSs associated with CFSs (Fig. 6C). To ensure that the low pG4 clusters coverage observed in long genes compared to shorter genes was not biased by pG4 clusters enrichment at TSSs, we reanalysed this coverage by removing the first 2 kb of the genes. This new analysis still shows lower coverage in long genes, confirming our initial observation (Fig. 6B).

Together, these results indicate that late-replicating domains are depleted in strong replication initiation signals. Gene bodies of CFSs, which are embedded within late-replicating domains, follow this overall trend. However, CFS-associated genes display increased accumulation of pG4 clusters at their TSSs relative to shorter genes.

## Discussion

**Figure 7.**
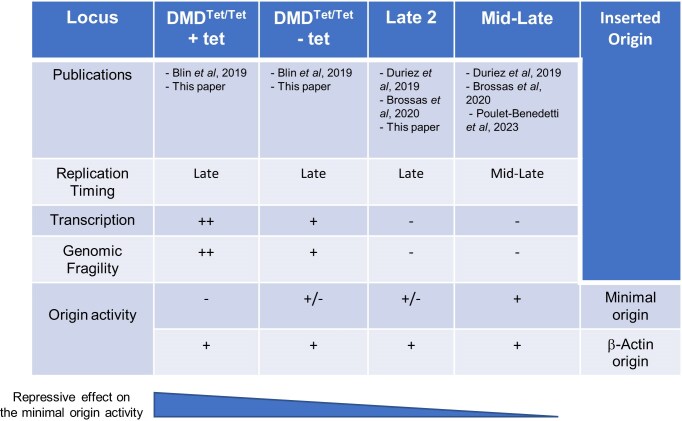
A summary of the results obtained from inserting the minimal and β-actin origins at three different loci. The publications describing the results relevant to this study are indicated in the top row. In the DMD^Tet/Tet^ cell line, induction with tetracycline leads to a two- to threefold increase in nascent RNAs and a fourfold increase in the % of broken chromosomes in metaphase. Origin activity was assessed using two assays: (i) the SNS relative enrichment and (ii) the origin’s ability to locally advance the RT. A ‘+’ indicates a high level of SNS relative enrichment and local RT advancement. A ‘±’ indicates a low SNS relative enrichment without local RT advancement. A ‘−’ indicates an SNS relative enrichment comparable to background and no detectable RT advancement.

Transcription and replication machineries share the same DNA template, making their coordination inherently complex. This complexity is heightened by the temporal separation between the assembly of pre-RCs and the initiation of replication. During the G1 phase, pre-RCs displaced by transcription can be reloaded dynamically [37]. However, once the S phase begins, new pre-RCs can no longer be assembled. Therefore, their displacement by transcription leads to a blockade of replication initiation within actively transcribed regions. Pre-RCs activated at the onset of S phase face only a short window during which transcriptional interference can occur, whereas those activated later in S phase are exposed to a much longer period of potential disruption. To investigate how transcription affects replication origins in late-replicating contexts *in vivo*, we compared a minimal 90 bp efficient origin devoid of promoter activity to a longer 1264 bp origin containing a functional promoter. Our results show that the chromosomal environment of a long, transcriptionally active, late-replicating gene corresponding to a CFS strongly represses the activity of the minimal efficient replication origin (Figs 5B and 7).

This observation aligns with previous findings from molecular combing studies, which have revealed a depletion of replication initiation events in the cores of CFSs, such as FRA3B in human cells and DMD in chicken cells, in a transcription-dependent manner. However, direct experimental evidence that transcription can inactivate an efficient replication origin has been lacking. A prior study in avian DT40 cells reported a reduction of initiation events in the first half of the transcribed DMD gene. Here, our work demonstrates that this depletion correlates with nascent transcription density, suggesting a direct relationship between transcriptional activity and repression of local origin firing. To further probe this relationship, we assessed the activity of an efficient minimal origin inserted into the transcriptionally depleted region under conditions that induce gene fragility. In the absence of tetracycline-induced transcription, the minimal origin remained functional, albeit repressed. However, upon induction, transcription inactivated the minimal origin. Notably, the repression of origin activity increased in tandem with elevated transcription and fragility of the DMD gene, pointing to a causal relationship. A previous study using the same inducible cell line showed that tetracycline treatment increased the proportion of chromosome breaks from 13% to 48% (Fig. 4) [21]. Our finding that origin inactivation accompanies this increase in fragility supports the notion that CFS instability results from the transcription-dependent suppression of replication initiation events by RNA polymerase II (Fig. 4). Interestingly, the strong β-actin promoter/origin remained efficient even under induced DMD transcription. This finding highlights a functional hierarchy among replication origins, with some origins capable of functioning independently of their chromatin or transcriptional environment. We observed that the β-actin origin contains multiple G-quadruplexes (pG4s), including three pG4 dimers, which may be essential for the formation of a robust origin. Origins of this type are likely well represented among the constitutive or core origins described in Picard *et al.* [38] and Akerman *et al.* [39], respectively. Previous work has shown that such pG4 dimers are critical for the activity of the minimal origin and are found in ~30% of efficient origins in both human and chicken cells [27]. However, we did not genetically dissect the 1264 bp β-actin origin to define which *cis*-elements are required for its function. Our findings suggest that such elements are depleted from CFSs, consistent with the AT-rich nature of these genomic regions. As predicted, the coverage of pG4 clusters along CFSs is lower than that found in smaller genes. However, our analysis showed that pG4s clusters are more enriched at the TSS of CFSs than those of smaller genes. This observation may suggest that a pressure of selection has operated to maintain efficient sites of replication initiation in the vicinity of CFSs to limit under-replication at these sites. In line with this hypothesis, it has been shown that pG4s located at promoters and replication origins are under selective pressure [40].

To better define the functional hierarchy among origins observed at the DMD CFS locus, we next examined the impact of a late-replicating chromatin context lacking transcriptional activity on the function of both the minimal and β-actin origins. Previously, we demonstrated that the minimal origin is efficient within a mid-late S phase region, as evidenced by strong relative enrichment of SNSs and a significant advance in local RT [27] (Fig. 7). In this context, the β-actin origin exhibited similar behaviour. However, in a silent, late-replicating region, the minimal origin shows modest SNS enrichment and induces no significant change in RT, reflecting reduced origin activity. In contrast, insertion of the β-actin origin into the same region resulted in a pronounced advance in RT, mirroring our results in the transcribed DMD gene. This supports the existence of a functional hierarchy among efficient origins, likely defined by the presence and arrangement of dimeric pG4 *cis*-elements and the capacity of the sequence to also drive transcription. Moreover, this hierarchy appears to be context-dependent: in a more permissive (mid-late) region, both types of origins perform similarly, whereas in a more restrictive (late-replicating) context, the minimal origin is repressed while the promoter-associated β-actin origin remains active. These results underline the critical role of *cis*-elements in the establishment of the spatiotemporal replication programme, while also highlighting the role of RNA polymerase II passage in the establishment of regions depleted in replication initiation events. In this study, we focused on a late-replicating, transcriptionally active region. Further investigations are required to assess the impact of the high transcriptional activity observed in early-replicating regions on replication origin firing. In addition to CFSs, early-replicating fragile sites constitute a distinct class of fragile genomic regions localized in highly transcribed, gene- and R-loops-rich domains that replicate early in S phase [41]. This organization may promote genotoxic head-on collisions between the DNA replication machinery and RNA polymerase [42]. Transcription-replication interference emerges as a fundamental mechanism driving fragile site formation throughout S phase, although the mechanisms involved in the early and late S phase are quite different. Therefore, exploring the behaviour of our minimal origin model in an early replicating region would provide a more comprehensive understanding of how transcriptional activity, RT, and the chromatin environment collectively influence origin function and genome stability.

While this study provides direct evidence of transcription-dependent origin repression, several aspects warrant consideration. First, genetic analyses were conducted using transcription driven by an artificial tetracycline-inducible promoter, which may influence the replication machinery differently from transcription initiated at endogenous CFS promoters. However, the genomic context of the DMD locus into which the constructs were inserted is homologous to a well-characterized human CFS and is therefore expected to recapitulate similar replication behaviour. Second, all genetic experiments were conducted in the avian DT40 cell line, in which CFS dynamics may differ from those observed in human cells. Nevertheless, mapping of fragile sites in this avian cell line identified regions homologous to established human CFSs that share key features, including long transcribed genes that replicate late. Notably, avian homologs of human CFS genes have retained their large intronic regions despite the global trend towards intron size reduction in birds [43]. This conservation, despite their fragility, suggests that CFS-associated genes achieve an evolutionarily conserved biological function that remains to be elucidated. Finally, the identification of similar *cis*-determinants of efficient replication origin activity in both chicken and human genomes supports the relevance of our findings to the mechanisms operating at human CFSs [27]. Third, the results are based on population-level analyses, so the effects of transcription on origin firing, and *vice versa*, in individual cells can only be inferred.

## Supplementary Material

gkag297_Supplemental_File

## Data Availability

Nascent RNA raw sequencing files and processed files generated in this study for data visualization have been deposited in Zenodo (https://zenodo.org/) and are accessible using the DOI 10.5281/zenodo.17456258. Processed BigWig files are viewable on the UCSC browser using the link: https://genome.ucsc.edu/s/Doncarli_Caroline/Replication_origin_hierarchy_at_fragile_sites.
