# Supplementary material for: Transcription at an inducible common fragile site reveals replication origin strength hierarchy
Source: Nucleic Acids Res. 2026 Apr 7;54(6):gkag297. doi: 10.1093/nar/gkag297 (PMC13062775; doi:10.1093/nar/gkag297)
Supplement: gkag297_Supplemental_File [file gkag297_supplemental_file.pdf]

Supplementary Information

Transcription at an inducible common fragile site reveals replication origin strength hierarchy

Juliette Mandelbrojt[1]†, Caroline Tonnerre-Doncarli[1] †*, Théo Baret[1], Aurélie Masson[1], Michelle Debatisse[2,3] and Marie-Noëlle Prioleau[1*]

Page 2 - 5: Supplementary Figures 1 - 4

Page 6 - 11: Supplementary Tables 1 - 6

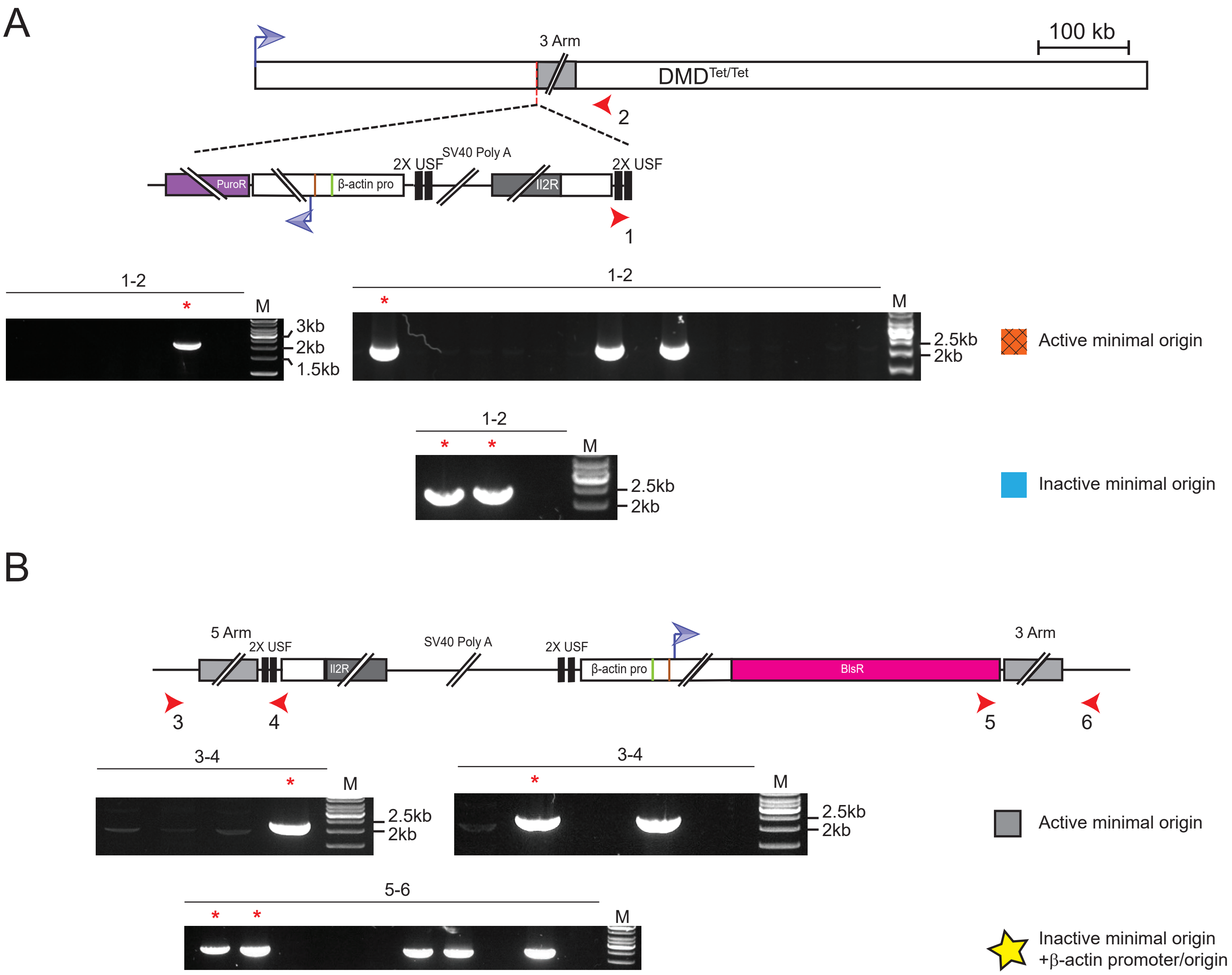

Supplementary Fig.1. PCR validation of clones selected for homologous recombination

(A-B) Schematic diagrams showing genomic regions containing a site-specific integrated construct. The 5′ and/or 3′ arms of the targeted vector are shown. Arrows indicate primers used to analyse the correct integration of the constructs by homologous recombination in the target region. PCR products were subjected to electrophoresis in a 1% w/v agarose gel and stained with SYBR safe. The DNA size marker is a commercial 1 kb DNA ladder (M). Lanes marked with a star correspond to clonal cell lines selected for further analysis. (A) $DMD^{Tet/Tet}$ locus insertion site containing the minimal active or inactive origin is shown. (B) Late 2 insertion site, containing the minimal active or inactive origin associated with the β-actin+BlsR construct, is shown.

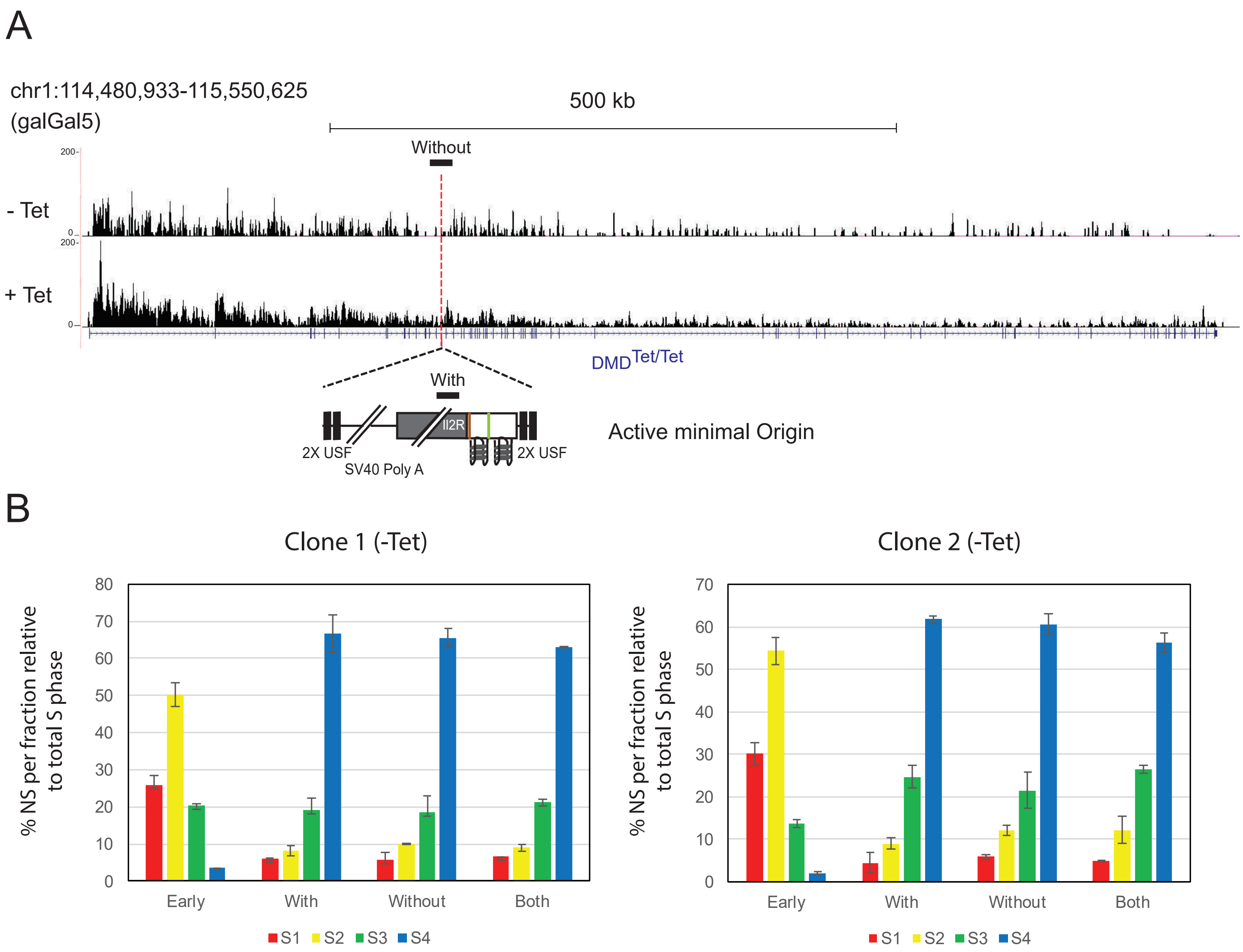

Supplementary Fig. 2.

(A) UCSC genome browser view showing the EU-seq signal across the $DMD^{Tet/Tet}$ gene carrying the active minimal origin on one allele in cells grown without and with tetracycline. The insertion site for the active minimal origin is indicated by a dotted line. Below schematic representations of the inserted construct is shown. Lines indicate amplicons used to distinguish the RT profile of the modified allele (With) and the WT allele (Without). (B) Replication timing (RT) profiles of each chromosomal allele were determined in the cell line described in A, using the allele-specific quantitative PCR analysis. The cells were grown in the absence of tetracycline before RT analysis. BrdU pulse-labelled cells were sorted into four S-phase fractions, from early to late (S1 to S4), and the immunoprecipitated newly synthesized DNA strands (NS) were quantified in each fraction by qPCR. Specific primer pairs were used to distinguish the RT profile of the modified allele (With), the WT allele (Without) and 5 kb away from the insertion site on both alleles (Both). The endogenous β-globin locus was analysed as an early-replicated control (Early).

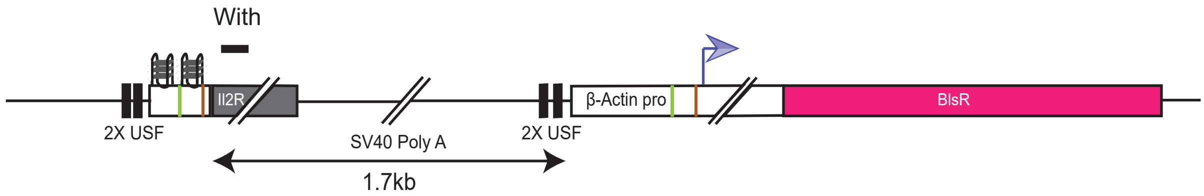

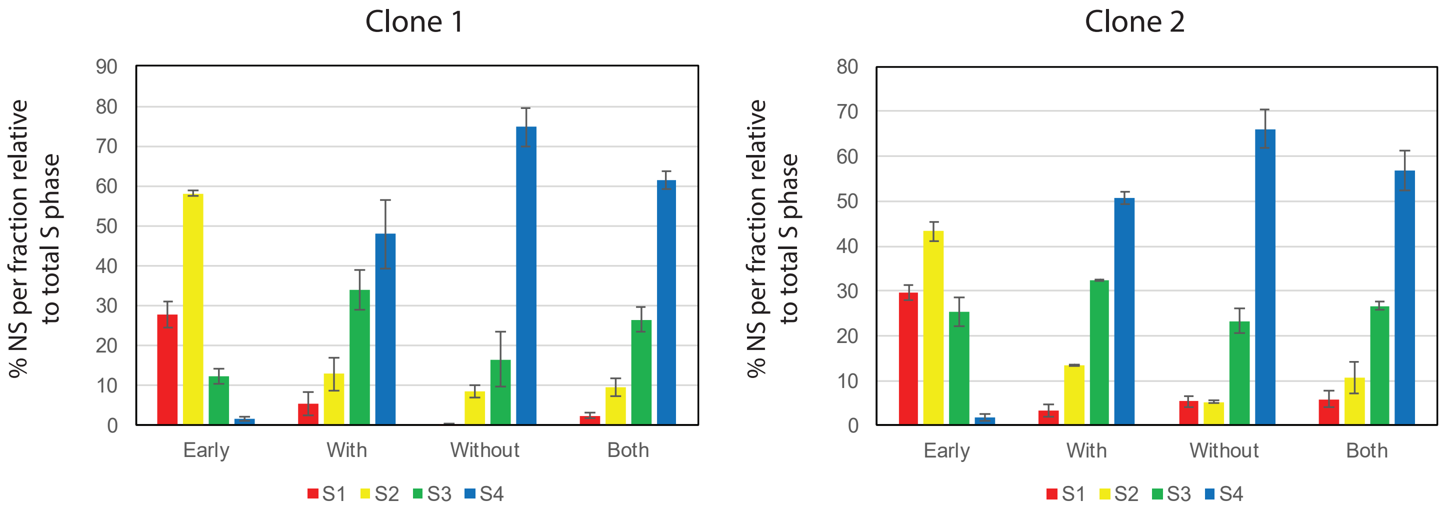

Supplementary Fig. 3.

Schematic representation of the analysed cell line. The construct was inserted at the Late2 locus (chr1:177,936,192 bp, galGal5) previously described in Duriez *et al.*, 2019, and Brossas *et al.*, 2020. The cell line harbours an active minimal origin fused to the β-actin promoter/origin. The line above the construct indicates the position of the with amplicon used for replication timing experiments. The replication timing (RT) profiles of each chromosomal allele were determined in the aforementioned cell line using the allele-specific quantitative PCR analysis. The cells were grown in the absence of tetracycline prior to RT analysis. BrdU pulse-labelled cells were sorted into four S-phase fractions, from early to late (S1 to S4), and the immunoprecipitated newly synthesized DNA strands (NS) were quantified by qPCR in each fraction. Specific primer pairs were used to distinguish the RT profile of the modified allele (With), the WT allele (Without) and 5 kb away from the insertion site on both alleles (Both). The endogenous β-globin locus was analysed as an early-replicated control (Early).

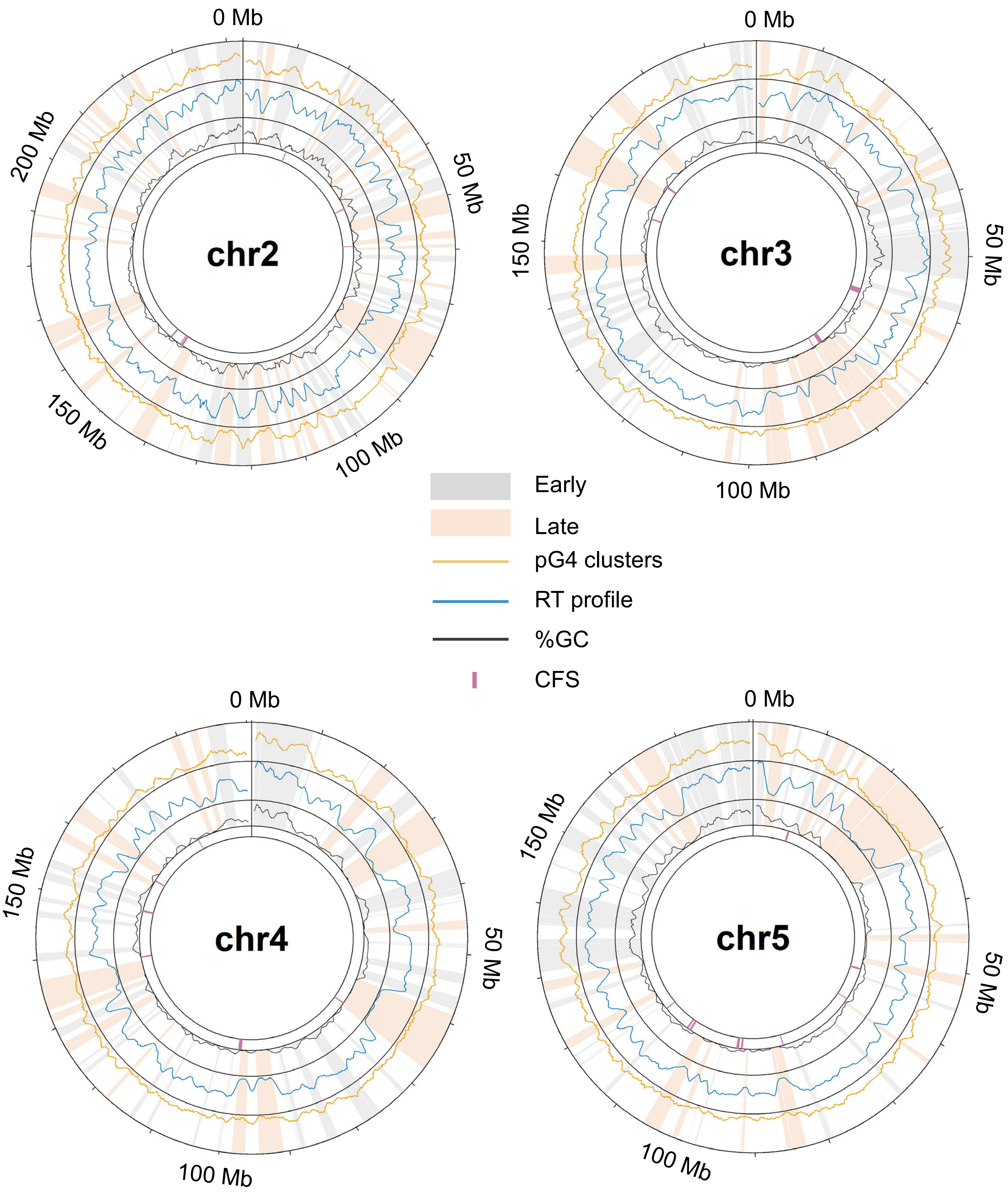

Supplementary Fig. 4

Circos plot showing from the outside in pG4 clusters coverage, replication timing profile, GC% and CFSs across human chromosome 2, 3, 4 and 5 (hg19). The top 20% earliest and latest replicating regions are highlighted in grey and orange, respectively.

Supplementary Table 1. Primer sets used for plasmids constructions and quantitative PCR

Primer name	Figure	Forward primer sequence	Reverse primer sequence	Position (Build Mars 2018)
IL2R (1) /With	1-4, Suppl. Fig. 2-3	GGGGACTGCTCACGTTCATCA	AATGTGGCGTGTGGGATCTC	
IL2R (2)	1, 2, 4	CTACACAGAGGTCCTGCTG	TGCCTGAGGCTTCTCTTCAC	
2xUSF (3')	1, 2, 4	TGCATTCTAGTTGTGGTTTGTCC	ACCGTCGACCAACTTTGTATAGA	
**CFS locus**				
β-actin	2	ACGTGCTGGTTATTGTGCTGT	TCGGTCATGGAAGGTCGTCTC	
2xUSF (5')	1	TGTGTAGTGACTGTACCTATGCTT	CCGTGCCACCTCAACTTTTGTAT	
Bkgd (CFS site) / Both	1 - 3, Suppl. Fig. 2	TGGATTTCCCAGCAGCTCTT	GCTTCCCAACCCCAACTTTT	chr1:116871770-116871835
Without	1 - 3, Suppl. Fig. 2	ACCATGCTAAGATGATGACTGTGA	TGTGAAGCTATGCAATGTTCCAGT	chr1:116877422+116877511
**Late locus**				
β-actin	4	TGCAGAAATCGGAGGAAGAAGA	GAATTGCCGCTCCCACATGA	
Bkgd (late site)	4	CGTCAGAGTGGTGGTGAGAA	TCTTGCCCAAACCAAAAACA	chr1:179238582+179238693
Both	4	CAAGGTTTCCACCCCTAAAGA	TGATGGATGTGGGAAGAGAAA	chr1:179246338+179246419
Wt allele / Without	4	TTTACACTACTCCCACCCCTCG	TTGACCATATGCCACCAACACC	chr1:179250225+179250324
**Controls**				
ρ-origin	1-3	GACGGTCAGGTTTGCCAAAG	TCCTGAGGATACGTTTTTCAG	chr1:197287850-197288114
Bu1a gene	1	AATGTCCCCAAAATGAGCTG	CCTCTTTTTCCACCCTCCTC	chr1:93337763+93337907
Early timing control	3, 4, Suppl. Fig. 2-3	GACGGTCAGGTTTGCCAAAG	TCCTGAGGATACGTTTTTCAG	chr1:197287850-197288114
Mitochondrial DNA	3, 4, Suppl. Fig. 2-3	CATCCCATGCATAACTCCTG	GTAGTCCAGGCTTCACTTGA	chrM:541+731
**Constructions**				
5 Arm for minimal origin+β-actin-PuroR insertion in DMDTetO		GGGGACAAGTTTGTACAAAAAAGCAG GCTTCCTGCCTCCAAGTCTACACA	GGGGACAACTTTTGTATACAAAGTTG CCTTACCTGCCCCAAACTGC	chr1:116875053+116877153
3 Arm for minimal origin+β-actin-PuroR insertion in DMDTetO		GGGGACAACTTTGTATAATAAAGTTGT GGAGCAACTACCCTGTCCT	GGGGACCACTTTGTACAAGAAAGCT GGGTCCACCCAACTTGCACACTGT	chr1:116879085+116881038
**screening of targetted integration**				
5'-screening-DMD gene for active minimal origin+β-actin-PuroR (1-2)	Suppl. Fig. 1	TACACATTCACAGAGGAAAAGA	GCCACCTCAACTTTTGTATAC	
5'-screening-late2 site for minimal active origin+β-actin-BlsR (3-4)	Suppl. Fig. 1	ACTTTGCACAAGCTAAGGAACC	AAAAGTTGAGGTGGCACGGG	
3'-screening-late2 site for minimal inactive origin+β-actin-BlsR (5-6)	Suppl. Fig. 1	TGCATTCTAGTTGTGGTTTGTCC	ATCTCTGCCTTCAAACCTTCAG	

Supplementary Table 2. Transgene copy number determination in clonal cell lines

The table shows the qPCR results obtained with genomic DNA extracted from the clones selected for the experiments. For each clone, 1 or 2 ng of genomic DNA was amplified with a primer set amplifying a sequence within the construct (With) and another primer set amplifying a sequence on both alleles (Both). The crossing point (Cp) was obtained by using the second derivative maximum method described in the LightCycler Software (Roche). Each primer pair has a PCR efficiency around 95%; therefore, the difference in the crossing point (Cp) between one and two copies should approximate one cycle. Analysis of transgene copy number shows that for most of the clones, a difference of ≥1.5 is observed between the primer pair With (one copy) and Both (two copies) due to slight variations in the efficiency of this primer pair that we have repeatedly observed. In any case this difference does not account for multiple copy insertion.

Locus	Cell line	Clone	Cp With	Cp Both	ΔCp
DMD	Active minimal origin	1	26.5	24.9	1.6
		2	26.5	24.9	1.6
	Inactive minimal origin	1	26.2	24.7	1.5
		2	26.3	24.8	1.5
	Inactive minimal origin + β-actin origin	1	25.2	23.9	1.3
		2	25.3	24.0	1.3
Late 2	Active minimal origin	1	26.3	24.8	1.5
		2	26.0	24.6	1.5
	Active minimal origin + β-actin origin	1	24.8	23.2	1.7
		2	25.3	23.7	1.6
	Inactive minimal origin + β-actin origin	1	25.5	23.8	1.7
		2	25.3	24.2	1.1
		3	25.6	24.3	1.3

Supplementary Table 3. Quantification of relative SNS enrichment and nascent RNAs in DMD$^{Tet/Tet}$ cell lines

(A) SNS relative enrichments along the ectopic active minimal origin obtained in clones (n=2, biological replicates) cultured without (-Tet) and with (+Tet) tetracycline or along the inactive minimal origin in clones (n=1) cultured without tetracycline (-Tet) presented in Figure 1C. The amplicons used for quantification are indicated in Fig. 1B. Bkgd refers to an amplicon located 5 kb away from the insertion site. (B) Relative quantification and Crossing point (Cp) values obtained for RT+-qPCR and RT$^-$-qPCR (background) experiments for relative quantification of nascent RNAs in the modified DMD$^{Tet/Tet}$ cell lines presented in Figure 1D, containing either the active or inactive minimal origin grown in the absence of tetracycline are reported in the table. NA indicates non-amplified signals. The amplicons used for quantification are shown in Figure 1B.

A

SNS relative enrichment (%)		2xUSF (3')	Il2R (2)	Il2R (1)	Bkgd
**Active Minimal Origin -Tet**	clone 1	0.87	23.65	39.58	3.18
		2.93	21.93	34.58	1.97
	clone 2	2.98	18.92	23.23	1.02
		2.69	13.26	20.74	0.36
**Active Minimal Origin +Tet**	clone 1	0.42	4.76	6.83	-
		1.56	6.63	5.81	0.24
	clone 2	0.64	3.15	5.05	0.22
		0.75	3.15	4.80	-
**Inactive Minimal Origin -Tet**	clone 1	0.75	1.23	2.22	0.49
		0.53	0.86	2.73	-

B

Relative quantification values RT+ qPCR

		Bu1a	*IL2R (2)*	*2xUSF (5')*	*Without*
Active Minimal Origin	clone 1	100	0.087	0.018	0.100
		100	0.096	0.020	0.111
		100	0.076	0.038	0.123
		100	0.085	0.042	0.136
	clone 2	100	0.066	0.016	0.072
		100	0.071	0.018	0.078
		100	0.087	0.018	0.063
		100	0.095	0.020	0.068
Inactive Minimal Origin	clone 1	100	0.093	0.035	0.129
		100	0.073	0.028	0.101
		100	0.107	0.040	0.109
		100	0.084	0.031	0.085
	clone 2	100	0.107	0.045	0.107
		100	0.064	0.027	0.064
		100	0.087	0.041	0.144
		100	0.052	0.025	0.086

Crossing point values RT+ qPCR

		Bu1a	*IL2R (2)*	*2xUSF (5')*	*Without*
Active Minimal Origin	clone 1	25.83	30.99	32.58	30.77
		25.98	31.19	31.48	30.47
	clone 2	26.03	31.64	32.95	31.5
		26.15	31.19	32.77	31.69
Inactive Minimal Origin	clone 1	26.85	31.85	33.61	31.21
		26.48	31.59	33.28	31.46
	clone 2	27.22	32.09	33.68	31.84
		26.46	32.49	33.97	31.43

Crossing point values RT- qPCR

		Bu1a	*IL2R (2)*	*2xUSF (5')*	*Without*
Active Minimal Origin	clone 1	37.62	37.55	NA	NA
	clone 2	NA	40.21	NA	NA
Inactive Minimal Origin	clone 1	45	NA	NA	NA
	clone 2	NA	NA	NA	NA

Supplementary Table 4. Quantification of relative SNS enrichment and replication timing by real-time PCR in allele-specific experiments DMD$^{Tet/Tet}$ cell lines

(A) SNS relative enrichments along the ectopic construct containing both the inactive minimal origin and the β-actin promoter/origin obtained in clones (n=2, biological replicates) cultured with (+Tet) tetracycline presented in Figure 2C. The amplicons used for quantification are indicated in Fig. 2A. Bkgd refers to an amplicon located 5 kb away from the insertion site.(B-D) These tables presents quantitative real-time PCR data from replication timing (RT) experiments detailed in Fig. 3B-C (B,C) and Supplementary Fig 2B (D). RT profiles of each chromosomal allele were determined after targeted transgene integration, using the allele-specific analysis by quantitative PCR. BrdU pulse-labelled cells were sorted into four S-phase fractions, from early to late (S1 to S4) and the immuno-precipitated newly synthesized strands were quantified by qPCR in each fraction. Specific primer pairs determine the RT profile for the modified allele (With), the WT allele (Without) and 5 kb (B-C) or 3.5 kb (D) away from the insertion site on both alleles (Both). The endogenous β-globin locus was analysed as an early-replicated control (Early). Two independent clones were analysed. 'exp.' denotes technical replicates of each RT assay. Cells were grown in the absence (-Tet, B) or in the presence (+Tet, C) of tetracycline for 24 hours before RT analysis.

A

SNS relative enrichment (%)		β-actin	2xUSF (3')	Il2R (2)	Il2R (1)	Bkgd
Inactive Minimal Origin +β-actin promoter/origin +Tet	clone 1	38.7	12.51	4.22	1.33	0.09
		46.0	17.17	4.77	1.74	0.27
	clone 2	52.1	22.43	7.94	5.04	0.35
		79.8	32.40	9.54	6.57	0.65

B

	Clone 1 -Tet					Clone 2 -Tet				
	%S phase	Early	With	Without	Both	%S phase	Early	With	Without	Both
exp. 1	S1	19.8	2.6	6.4	3.4	S1	22.1	2.7	2.1	2.6
	S2	62.4	20.2	8.8	14.1	S2	50.4	12.1	6.5	11.4
	S3	15.7	45.4	23.5	41.7	S3	25.6	33.2	19.3	28.2
	S4	2.1	31.8	61.3	40.8	S4	1.9	52.1	72.1	57.7
exp. 2	S1	17.3	2.4	5.3	3.5	S1	17.1	0.7	2.8	2.3
	S2	62.9	16.2	9.4	13.0	S2	45.5	9.1	3.1	8.6
	S3	18.1	51.4	28.5	44.3	S3	35.8	43.5	22.5	38.1
	S4	1.7	30.0	56.8	39.2	S4	1.6	46.6	71.6	51.0
exp. 3	S1	20.3	4.9	-	3.2	S1	18.8	1.5	2.3	2.3
	S2	61.4	18.3	-	14.2	S2	50.4	9.2	4.7	10.6
	S3	14.9	45.5	-	42.2	S3	29.1	36.2	20.8	40.7
	S4	3.5	31.3	-	40.4	S4	1.6	57.3	72.2	46.3

C

	Clone 1 +Tet					Clone 2 +Tet				
	%S phase	Early	With	Without	Both	%S phase	Early	With	Without	Both
exp. 1	S1	27.76	2.48	2.85	3.45	S1	24.9	2.2	2.4	2.3
	S2	50.52	13.63	10.36	10.11	S2	52.0	15.4	10.8	9.9
	S3	19.37	34.75	15.79	29.44	S3	20.6	34.5	18.9	35.4
	S4	2.35	49.15	70.99	57.01	S4	2.5	48.0	67.9	52.4
exp. 2	S1	25.5	2.4	2.0	2.9	S1	22.6	2.2	2.5	1.7
	S2	50.4	16.8	6.3	10.7	S2	54.9	16.4	6.7	13.2
	S3	20.7	32.9	17.3	27.8	S3	20.2	36.5	17.0	34.6
	S4	3.4	47.8	74.4	58.6	S4	2.3	44.9	73.8	50.5

D

	Clone 1 -Tet					Clone 2 -Tet				
	%S phase	Early	With	Without	Both	%S phase	Early	With	Without	Both
exp. 1	S1	27.72	6.17	4.44	6.64	S1	28.22	1.74	5.53	4.94
	S2	48.03	9.32	10.10	8.33	S2	56.55	9.36	11.29	14.48
	S3	20.69	21.48	21.61	21.88	S3	13.06	27.51	24.46	25.90
	S4	3.55	63.04	63.86	63.15	S4	2.17	61.39	58.72	54.68
exp. 2	S1	23.97	5.80	7.23	6.57	S1	32.02	5.11	6.24	4.91
	S2	52.49	7.33	10.06	9.66	S2	52.14	7.47	12.95	9.95
	S3	20.02	16.67	15.37	20.88	S3	14.24	24.63	18.57	27.23
	S4	3.53	70.19	67.33	62.89	S4	1.60	62.79	62.24	57.92
exp. 3						S1	-	6.44	-	-
						S2	-	10.06	-	-
						S3	-	22.05	-	-
						S4	-	61.44	-	-

Supplementary Table 5. Quantification of relative SNS enrichment and replication timing in allele-specific experiments by real-time PCR in Late2 cell lines

(A) SNS relative enrichments along the ectopic active minimal origin or the inactive minimal origin combined with the β-actin origin obtained in two independant clones (n=2, biological replicates) presented in Figure 4B. The amplicons used for quantification are indicated in Fig. 4A. Bkgd refers to an amplicon located 11 kb away from the insertion site. (B-C) These tables presents quantitative real-time PCR data from replication timing (RT) experiments detailed in Fig. 4C-D. RT profiles of each chromosomal allele were determined after targeted transgene integration, using the allele-specific analysis by quantitative PCR. BrdU pulse-labelled cells were sorted into four S-phase fractions, from early to late (S1 to S4) and the immuno-precipitated newly synthesized strands were quantified by qPCR in each fraction. Specific primer pairs determine the RT profile for the modified allele (With), the WT allele (Without) and 3.5 kb away from the insertion site on both alleles (Both). The endogenous β-globin locus was analysed as an early-replicated control (Early). Two independent clones were analysed. 'exp.' denotes technical replicates of each RT assay.

A

SNS relative enrichment (%)		Il2R (1)	Il2R (2)	2xUSF (3')	β-actin	Bkgd
Active Minimal Origin	Clone 1	28.33	10.37	2.25	-	0.27
		31.40	10.82	2.11	-	0.39
	Clone 2	20.41	5.83	0.63	-	0.25
		16.68	4.87	1.34	-	0.21
Inactive Minimal Origin + βactin Origin	Clone 1	4.29	4.95	15.37	66.29	0.27
		4.12	4.72	14.35	69.77	0.62
		-	-	16.93	83.47	-
		-	-	13.67	78.29	-
		-	-	18.14	65.86	-
		-	-	13.96	74.86	-
		-	-	10.89	52.98	-
		-	-	-	52.98	-
	Clone 2	6.10	5.63	15.77	45.89	0.45
		6.48	5.67	19.53	44.45	0.40
		-	-	16.55	54.55	-
		-	-	16.10	48.30	-
		-	-	16.10	84.84	-
		-	-	16.55	32.27	-
		-	-	18.74	33.54	-

B

	Clone 1				
	%S phase	Early	With	Without	Both
exp. 1	S1	33.02	2.15	2.60	4.58
	S2	51.56	3.09	2.45	3.57
	S3	12.91	16.95	13.81	12.99
	S4	2.50	77.80	81.14	78.86
exp. 2	S1	33.02	1.01	1.68	3.61
	S2	49.66	1.74	2.42	5.54
	S3	15.15	13.03	16.43	15.40
	S4	2.18	84.22	79.47	75.45

	Clone 2				
	%S phase	Early	With	Without	Both
exp. 1	S1	45.17	3.42	2.76	4.12
	S2	43.63	8.14	4.91	7.23
	S3	6.39	20.63	20.45	19.13
	S4	4.80	67.81	71.89	69.52
exp. 2	S1	51.58	1.83	3.78	5.34
	S2	36.57	4.51	5.05	9.24
	S3	7.59	11.32	24.30	19.91
	S4	4.26	82.34	66.87	65.51

C

	Clone 1				
	%S phase	Early	With	Without	Both
exp. 1	S1	27.01	1.99	0.75	2.32
	S2	48.70	9.57	3.38	6.01
	S3	22.50	50.52	28.31	37.25
	S4	1.79	37.92	67.56	54.42
exp. 2	S1	34.75	3.32	4.05	3.70
	S2	51.04	15.55	4.20	10.1
	S3	12.44	41,.4	13.92	28.70
	S4	1.77	39.79	77.83	56.89

Supplementary Table 6. Quantification of replication timing in allele-specific experiments by real-time PCR in Late2 cell lines

These tables presents quantitative real-time PCR data from replication timing (RT) experiments detailed in Suppl. Fig 3. RT profiles of each chromosomal allele were determined after targeted transgene integration, using the allele-specific analysis by quantitative PCR. BrdU pulse-labelled cells were sorted into four S-phase fractions, from early to late (S1 to S4) and the immuno-precipitated newly synthesized strands were quantified by qPCR in each fraction. Specific primer pairs determine the RT profile for the modified allele (With), the WT allele (Without) and either 5kb away from the insertion site on both alleles (Both). The endogenous β-globin locus was analysed as an early-replicated control (Early). Two independent clones were analysed. 'exp.' denotes technical replicates of each RT assay.

	Clone 1					Clone 2				
	%S phase	Early	With	Without	Both	%S phase	Early	With	Without	Both
exp. 1	S1	25.59	2.68	0.05	-	S1	28.44	2.47	6.18	7.19
	S2	58.60	13.40	7.39	-	S2	41.80	13.52	5.47	13.02
	S3	13.73	30.67	21.37	-	S3	27.52	32.36	25.22	26.03
	S4	2.08	53.24	71.19	-	S4	2.24	51.65	63.12	53.76
exp. 2	S1	30.13	2.97	0.35	1.74	S1	30.76	4.23	4.52	4.61
	S2	57.74	9.73	9.71	12.03	S2	44.74	13.37	4.98	8.10
	S3	10.88	28.03	11.75	24.48	S3	23.17	32.57	21.41	27.39
	S4	1.25	59.26	78.19	61.75	S4	1.33	49.83	69.09	59.90
exp. 3	-	-	8.37	-	2.93					
	-	-	13.92	-	7.88					
	-	-	38.11	-	29.95					
	-	-	39.60	-	59.25					
exp. 4	-	-	8.70	-	2.59					
	-	-	18.55	-	8.71					
	-	-	32.51	-	25.04					
	-	-	40.24	-	63.66					
exp. 5	-	-	4.34	-	-					
	-	-	8.48	-	-					
	-	-	40.15	-	-					
	-	-	47.03	-	-					
